# The State of the Art of the DSM-5 “with Mixed Features” Specifier

**DOI:** 10.1155/2015/757258

**Published:** 2015-08-25

**Authors:** Norma Verdolini, Mark Agius, Laura Ferranti, Patrizia Moretti, Massimiliano Piselli, Roberto Quartesan

**Affiliations:** ^1^School of Specialization in Psychiatry, Division of Psychiatry, Clinical Psychology and Rehabilitation, Department of Medicine, University of Perugia, Piazzale Lucio Severi 1, Edificio A, 06132 Perugia, Italy; ^2^Santa Maria della Misericordia Hospital, Perugia, Italy; ^3^Department of Psychiatry, East London Partnership Foundation Trust, Bedfordshire Centre for Mental Health Research in Association with the University of Cambridge, University of Cambridge, Charter House, Alma Street, Luton LU1 2PJ, UK; ^4^Clare College Cambridge, The University of Cambridge, Cambridge, UK; ^5^Biotechnologies and Biomaterials in Vascular and Metabolic-Endocrine Diseases, University of Perugia, Piazzale Lucio Severi 1, Edificio A, 06132 Perugia, Italy; ^6^Division of Psychiatry, Clinical Psychology and Rehabilitation, Department of Medicine, University of Perugia, Piazzale Lucio Severi 1, Edificio A, 06132 Perugia, Italy; ^7^Functional Area of Psychiatry, University of Perugia, Piazzale Lucio Severi 1, Edificio A, 06132 Perugia, Italy; ^8^AUSL Umbria 2, Servizio Psichiatrico Diagnosi e Cura, Ospedale S. Giovanni Battista, Via Massimo Arcamone, 06034 Foligno, Italy

## Abstract

The new DSM-5 “with mixed features” specifier (MFS) has renewed the interest of the scientific community in mixed states, leading not only to new clinical studies but also to new criticisms of the current nosology. Consequently, in our paper we have reviewed the latest literature, trying to understand the reactions of psychiatrists to the new nosology and its epidemiological, prognostic, and clinical consequences. It seems that the most widespread major criticism is the exclusion from the DSM-5 MFS of overlapping symptoms (such as psychomotor agitation, irritability, and distractibility), with a consequent reduction in diagnostic power. On the other hand, undoubtedly the new DSM-5 classification has helped to identify more patients suffering from a mixed state by broadening the narrow DSM-IV-TR criteria. As for the clinical presentation, the epidemiological data, and the therapeutic outcomes, the latest literature does not point out a univocal point of view and further research is needed to fully assess the implications of the new DSM-5 MFS. It is our view that a diagnostic category should be preferred to a specifier and mixed states should be better considered as a spectrum of states, according to what was stated many years ago by Kraepelin.

## 1. Introduction

The current notion of mood disorders is based on the contrast between two most important concepts: on the one hand, Kraepelin [1], together with his famous successor Weygandt, considered all recurrent mood states, whether depressive or manic, as one illness, manic-depressive insanity, and thought that mixed states were the most common version of manic-depressive illness [2]. On the other hand, Leonhard distinguished patients with unipolar disorder from those with bipolar disorder on the basis of genetic and course findings, leading to the bipolar/unipolar dichotomy [3].

The DSM nosology of mood is neo-Leonhardian and not Kraepelinian [4]. Consequently, mixed states, as an overlapping of manic and depressive symptoms, have been almost completely neglected for decades, mainly under the influence of Kurt Schneider who promoted the idea that what may give the impression of the combination of manic and depressive features actually should not be considered a mood disorder [5].

A renewed interest in mixed states has developed after the new DSM-5 “with mixed features” specifier (MFS), even though the latest literature does not point out a univocal point of view [6] on the validity and utility of this new classification for mixed states. Some authors [7] suggest that it would capture subthreshold nonoverlapping symptoms of the opposite pole; others consider it to lead to more misdiagnosis and inadequate treatment [4]. Consequently, there is pressing need to validate the DSM-5 MFS in further research [8].

But what is the state of the art of the DSM-5 MFS? In order to give an answer to this question, we reviewed the latest literature on mixed states, in consideration of the new MFS in DSM-5, trying to understand the reactions of the psychiatric scientific community to the new nosology and its epidemiological, prognostic, and clinical consequences.

## 2. Materials and Methods

An electronic search using the MEDLINE database of published peer-reviewed papers was conducted in order to find the most up-to-date studies about mixed states according to the new DSM nosology, considering those published between January 2011 and February 2015. We used two groups of key words linked with the word AND: (1) bipolar mixed features, depressive mixed features, mixed features, mixed states; (2) DSM-5. We excluded studies published in other languages than English, “grey literature,” letters to the editor, studies about a paediatric population, and those using DSM-IV-TR criteria for mixed states.

## 3. Results

In our electronic search we found 37 papers and included in our review 19 papers according to our exclusion criteria. We decided to divide the papers into two main categories: (1) reviews and opinions about mixed states in DSM-5 (*n* = 9); (2) research reports (*n* = 11), with three subcategories: (2.1) clinical research reports (*n* = 5); (2.2) research reports about pharmacological treatment in the presence of MFS (*n* = 3); (2.3) research reports about diagnostic tools for mixed states (*n* = 2). The papers considered in the review are listed as follows, divided according to the specific categories.

*Summary of the Papers Considered in the Review, Divided according to Specific Categories*

1. Reviews and opinions about mixed states in DSM-5:
2. Koukopoulos et al., 2013 [4],
3. Koukopoulos and Sani, 2014 [9],
4. Uher et al., 2014 [10],
5. Perugi et al., 2014 [5],
6. Vieta and Valentí, 2013 [7],
7. de Dios et al., 2014 [11],
8. Liu and Jiang, 2014 [12],
9. Castle, 2014 [13],
10. First, 2011 [14].
11. Research reports:
  (2.1) clinical research reports:
   McIntyre et al., 2015 [15],
   Malhi et al., 2014 [16],
   Vieta et al., 2014 [17],
   Perlis et al., 2014 [18],
   Takeshima and Oka, 2015 [19],
  (2.2) research reports about pharmacological treatment in the presence of MFS:
   McIntyre et al., 2013 [8],
   Tohen et al., 2014 [20],
   Tohen et al., 2014 [21],
  (2.3) research reports about diagnostic tools for mixed states:
   Hergueta and Weiller, 2013 [22],
   Zimmerman et al., 2014 [23].

### 3.1. Reviews and Opinions about Mixed States in DSM-5

According to Koukopoulos and colleagues [4], the new DSM-5 MFS can lead to more problems in diagnosis, mainly in mixed depression. In fact, the DSM-5 task force decided to exclude from the MFS those manic and depressive symptoms that can overlap, leading to the exclusion of psychomotor agitation, irritability, and distractibility.

In an empirical study [9] the same authors found out that the frequency of mixed-mood states similar to the DSM-5 definition ranged from 0 to 12%. On the contrary, using a definition including those overlapping symptoms as central features of mixed depression, the frequency ranged from 33 to 47% [4].

It is for this reason that the authors [4] proposed the traditional name of “agitated depression” for depressive syndromes with psychomotor agitation and that the name “mixed depression” can be used in the absence of psychomotor agitation.

Furthermore, Uher and colleagues [10], when considering the implications for clinical practice and research of the changes between DSM-IV-TR and DSM-5, pointed out that the symptom of psychomotor agitation has been deleted from the MFS but added in the “with anxious distress” DSM-5 specifier in the fourth grade (“severe”), with the possibility of more misdiagnosis. In addition, they pointed out that irritability, which is seen in up to 40% of outpatients with major depressive disorder (MDD), contributes to episode severity and predicts recurrence but is not included among criteria for MDD in adults, not even in the MFS.

In addition, Perugi and colleagues [5] reported that even though the DSM-5 definition is based on the speculative wish to avoid “overlapping” manic and depressive symptoms, this combinatory model seems more appropriate for less severe forms, in which mood symptoms are clearly identifiable.

Another major criticism that Koukopoulos and colleagues [9] have made to the new DSM-5 MFS was that those hypo/manic symptoms required in a depressive episode to consider the MFS (i.e., elevated/expansive mood or grandiosity) are the least common symptoms that actually arise in depressive mixed states.

In 2013 Vieta and Valentì [7] published an enthusiastic review about mixed states in DSM-5. They stated that the new classification would capture subthreshold nonoverlapping symptoms of the opposite pole and would have a substantial impact in several fields, mainly because it would overcome the extremely narrow definition in DSM-IV-TR. In fact, fewer symptoms (than in DSM-IV-TR) of the opposite polarity are included in the MFS and the definition can be applied in more groups of disorders, not only in bipolar disorder I-BDI but also in bipolar disorder II-BDII, bipolar disorder not otherwise specified (BD-NOS), and, in particular, MDD, which is a link to the mood spectrum concept of Akiskal [24]. However, in the conclusions, the authors stated that the specificity and sensitivity of this diagnostic construct would need to be assessed by new and additional empirical studies.

In fact, the same group [11] published in the following year a review about bipolar disorders in DSM-5 focused on the fact that the decision to exclude common symptoms such as irritability, distractibility, or psychomotor agitation in spite of recognising the need to change the previous DSM-IV-TR strict criteria has been criticised as not very scientific and as lacking validity. Furthermore, they pointed out that the mixed nature of affective episodes became a course specifier with the disappearance of the category of mixed episode in bipolar disorders. In addition, the authors reported that agreement in diagnosis based on separate interviews by physicians who received training in the use of DSM-5 is moderate for bipolar disorder type (kappa: 0.56) and somewhat less for type II (kappa: 0.40) [11] and is the lowest ever reported for the MDD diagnosis (kappa: 0.28) [10].

Liu and Jiang [12] believed that one of the reasons for this could be the blurring of the depressive/bipolar disorder boundary.

Castle [13] stated that a major problem with the mixed states literature is the lack of uniform ways of measuring these syndromes and reported two examples of scales, one by Cavanagh et al. [25] comprising 18 items divided into an A group of manic items and a B group of depressive items. The author considered this scale of clinical utility because it is brief but requires validation in a depressed sample. The Multidimensional Assessment of Thymic States (MAThyS) was instead proposed by Henry et al. [26] and rated two dimensions, namely, activation/inhibition and emotional reactivity. The former was seen to be associated only with depression and the latter with manic and mixed symptoms.

Finally, according to the cost-benefit analysis drawn up by First [14], the new DSM-5 MFS is at odds with the previous empirical data and its overall treatment implications are also unclear. It was suggested that, by raising clinicians' awareness of the presence of mixed features through the introduction of this specifier, pressure will be exerted to do something with this information but without empirical evidence as a guide, it was not clear whether the possible interventions would do more good than harm. 

### 3.2. Research Reports

#### 3.2.1. Clinical Research Reports

In 2015, McIntyre and colleagues [15] published the results of The International Mood Disorders Collaborative Project (IMDCP). A total of 982 individuals who met criteria for a current mood episode as part of MDD (*n* = 506) or BD (*n* = 346; BDI: *n* = 216, BDII: *n* = 130) were included in the analysis and the DSM-5 MFS was evaluated using proxies by means of the Young Mania Rating Scale (YMRS) or the Montgomery Asberg Depression Rating Scale (MADRS) or the Hamilton Depression Rating Scale-17 items (HAMD-17). The authors found that 26.0% (*n* = 149) of patients diagnosed with a MDD, 34.0% (*n* = 65) of BDI patients, and 33.8% (*n* = 49) of individuals with BDII met the criteria for MFS during a mood depressive episode (MDE). On the contrary, MFS during a hypo/manic episode was identified in 20.4% (*n* = 52) of BDI participants and in 5.1% (*n* = 8) of individuals suffering from BDII.

Malhi and colleagues [16] conducted a study in order to examine the relative distribution of psychomotor agitation and distractibility in 200 patients divided into four groups: BD, BDspectrum, and unipolar depression (UP) and mixed depression (UPmix). The study was based on a self-report structured diagnostic assessment and a clinical psychiatric evaluation. The authors found that increased distraction, racing thoughts, and increased irritability were the most commonly reported manic symptoms amongst the UP group (*n* = 24, 17.6%). Furthermore, UPmix and BDspectrum groups had significantly higher psychomotor agitation (F (3, 122) = 8.04, *P* < 0.001) than the other two groups and distractibility was more represented in the UPmix (71%) and BDspectrum (67%) than in the UP (54%) and BD (40%) groups.

As for hypo/manic episodes with mixed features, in 2013 Vieta and colleagues [17] published the results of a multicenter, international online survey (the IMPACT study) conducted in order to describe the phenomenology of mania and depression in bipolar patients experiencing a manic episode with mixed features. Seven hundred patients with a manic episode with or without DSM-5 criteria for mixed features from 7 countries completed a 54-item questionnaire on demographics, diagnosis, symptomatology, communication of the disease, impact on life, and treatment received. Data was collected between March 26 and July 31, 2012. The authors found that 39% (*n* = 275) of patients reported ≥3 DSM-5 depressive symptoms during a past manic episode. These patients were more likely to have had a delay in diagnosis, were more likely to have experienced shorter symptom-free periods, and were characterized by a marked lower prevalence of typical manic manifestations. Indeed, only physical aggression and abusive behaviour towards others were more frequently reported by individuals in the mania with depressive symptoms group (*P* = 0.001 and *P* = 0.02). Furthermore, the authors also recorded a high rate of misdiagnosis (39% in the case of mania with depressive symptoms group); in particular these patients were significantly more frequently misdiagnosed as having insomnia than those without depressive symptoms (46.7% versus 27.9%; *P* < 0.0001).

As for MDE with mixed features, Perlis and colleagues [18] examined outcomes between patients with MDD participating in the Sequenced Treatment Alternatives to Relieve Depression (STAR*∗*D), hypothesizing that mixed symptoms in MDD would be associated with poorer antidepressant treatment outcomes. 9.6% (*n* = 231) of patients reported three or more mixed symptoms. The presence of such symptoms, in particular expansive mood and cheerfulness, was associated with a greater likelihood of remission (adjusted hazard ratio 1.16, 95% confidence interval 1.03–1.28). The authors concluded that this challenges the view that mixed features may be associated with poorer outcomes in MDD and questioned the notion that these features are or are not to be considered to be in the “bipolar spectrum.”

Takeshima and Oka [19] reported the data of 217 patients with MDE (BDII: *n* = 57, BD-NOS: *n* = 35, MDD: *n* = 125) firstly diagnosed according to DSM-IV-TR; at a later stage, the authors analyzed the prevalence of mixed depression according to both the DSM-5 MFS and Benazzi's definition (which includes all manic/hypomanic symptoms in MDE). The aim of the study was to assess if the lack of the three symptoms, irritability, psychomotor agitation, and distractibility, in the DSM-5 MFS could cause underdiagnosis of mixed depression. The authors identified more patients with Benazzi's mixed depression and DSM-5 defined mixed features in BD than in MDD (46.7% versus 12.8%, *P* < 0.0001, resp., according to Benazzi; 4.3% versus 0%, *P* = 0.0208, resp., according to DSM-5). Furthermore, they stated that the sensitivity/specificity for BD diagnosis according to Benazzi's mixed depression and DSM-5 defined mixed features were 55.1%/87.2% and 5.1%/100%, respectively.

#### 3.2.2. Research Reports about Pharmacological Treatment in the Presence of MFS

McIntyre and colleagues [8] described the frequency of depressive mixed features in BDI patients with manic episode by using MADRS and PANSS items as proxies for the DSM-5 MFS. They found that 34.2% (*n* = 328), 17.5% (*n* = 168), and 4.3% (*n* = 41) of the patients had at least 3 MADRS items with mild, moderate, and severe symptoms, respectively. Furthermore, the authors randomized the patients to asenapine, placebo, or olanzapine, showing that the symptomatic improvement of the manic/hypomanic symptoms was significant and superior to placebo for both asenapine and olanzapine, but for the former this was true regardless of depressive symptom severity, while on the contrary, for the latter the statement was true only in individuals with lower baseline depressive symptom severity.

Tohen and colleagues [20] investigated the correlations between the efficacy of olanzapine monotherapy (evaluated by change in MADRS total score from baseline to 6 weeks) and the number of concurrent manic symptoms (as measured by a Young Mania Rating Scale item score ≥1) in patients treated for bipolar depression. The percentage of patients in mixed feature ≥3 categories was 30.5% by using the 11 YMRS items and 11.9% by using the 6 YMRS items. Due to the fact that the least-square mean differences in MADRS total score and the effect sizes were similar when the authors considered 0, 1, or 2 and ≥3 mixed symptoms, they concluded that olanzapine worked similarly on bipolar depression irrespective of the number of concurrent manic symptoms.

The same authors published in a following paper [21] data about the comparison of the efficacy of olanzapine (evaluated by changes in the baseline-to-3-week YMRS total score) in patients with bipolar mania with or without DSM-5 mixed features (as determined by HAMD-17-item score ≥1). Sixty-six patients in the placebo group and 59 in the olanzapine group showed ≥3 mixed symptoms, in total 28% of the sample. Contrary to what happened for bipolar depression [20], olanzapine was efficacious in the treatment of bipolar I mania, in both patients without and with mixed features, but greater efficacy was seen in patients with mixed features who had more severe depressive symptoms (with mixed features effect size = 0.34; without mixed features effect size = 0.20).

#### 3.2.3. Research Reports about Diagnostic Tools for Mixed States

Hergueta and Weiller [22] reported the results of a validation study about a new module of the Mini International Neuropsychiatric Interview (M.I.N.I.) developed as a patient-completed questionnaire in order to evaluate the DSM-5 MFS for hypo/manic episodes. The study was conducted in two phases; the first one involved verification of bipolar patients' acceptance and comprehension of the questions in the module; the second consisted in the assessment of the degree of agreement between patients' responses using the M.I.N.I. module versus DSM-5 criteria as evaluated by psychiatrists. First of all, the authors reported that according to psychiatrists 46.5% (46/99) of patients met the DSM-5 MFS criteria but patients were more likely than psychiatrists to report the presence of at least three depressive symptoms (58.6%, 58/99). As for the agreement, it was substantial (Cohen's kappa coefficient = 0.60), the overall sensitivity of the M.I.N.I. was 0.91; and its specificity was 0.70. The module's positive and negative predictive values were 0.72 and 0.90. Interestingly enough, the highest levels of agreement were for the most common symptom (depressed mood, 79%) and the most severe symptom (suicidal thoughts, 85%), and the lowest level of agreement was for the least severe symptoms (i.e., fatigue, 64% to 65%). As a consequence, the authors considered this tool useful in clinical and epidemiological research and suggested that it should be incorporated into routine psychiatric evaluation of patients with manic episodes and that a corresponding module should be developed for evaluating “hypomanic features” during bipolar or unipolar major depressive episodes.

In fact, in 2014 Zimmerman and colleagues [23] modified their previously published depression scale (Clinically Useful Depression Outcome Scale (CUDOS)) to include a subscale assessing the DSM-5 MFS (CUDOS-M) in order to acknowledge the clinical significance of manic features in depressed patients. The CUDOS-M demonstrated excellent internal consistency (Cronbach's *α* = 0.84), the test-retest reliability of the total scale was high (*r* = 0.72), and the test-retest reliability of each item was significant (mean *r* = 0.56) though 3 items had a test-retest reliability of less than 0.30; consequently, the authors stated that repeated administration of such a measure during the course of treatment could enable clinicians to identify patients whose depressive episodes are evolving into a mixed state more readily and quickly.

## 4. Discussion

In our review, the percentage of hypo/manic episodes with mixed features ranges from 4.3% [8] to 58.6% [22]. The percentage of MDE with mixed features is lower and ranges from 0 [9, 19] to 34% [15]. These extremely broad ranges can be due to the quite low sensitivity of the DSM-5 MFS (5.1%) [19].

In fact, some authors suggested that DSM-5 has lowered the threshold for mixed states by limiting the symptoms that confer mixed features (psychomotor agitation, irritability, and distractibility), losing the essence of the clinical presentation [16].

Malhi et al. [16] stated that the exclusion of these symptoms would alter the trajectory of research and the implications of future findings.

According to this, in the research report published by Vieta and colleagues [17] more patients in the mania with depressive symptoms group had symptoms of anxiety and irritability associated with agitation than those without depressive symptoms (72.4% versus 27.1%; *P* < 0.0001). Furthermore, in Takeshima and Oka's study [19] psychomotor agitation was the manic/hypomanic symptom that was observed most frequently in both patients with BD (59.8%) and those with MDD (48.8%).

In the M.I.N.I. module for the DSM-5 MFS validation [22] psychomotor retardation was found to be the depressive symptom that patients presented least frequently during a hypo/manic episode.

The previous findings are in line with previous researchers' belief [27] that psychomotor agitation and distractibility are symptoms of particular interest because both have been identified as putative drivers in mixed episodes and are associated with poorer treatment outcomes [16, 28].

However, undoubtedly the new DSM-5 classification has helped to identify more patients suffering from a mixed state than the previous nosology and this may have clinical consequences. In fact, in McIntyre and colleagues [15] study, hypo/mania with MFS was identified in 20.4% (*n* = 52) of BDI participants of which 12.9% (*n* = 33) met criteria for a DSM-IV-TR defined mixed episode; this means that 7.5% (*n* = 19) of patients in this sample were considered not to have a mixed episode according to the previous nosology. In another study conducted by the same authors [8] between 20 and 40% (depending on symptom severity) of patients meeting criteria for DSM-5 defined mixed specifier did not meet DSM-IV-TR defined mixed features. Only Takeshima and Oka [19] found that the prevalence of DSM-5 MFS (4.3%) in the BD sample was lower than that of cooccurrence of MDE and HME (comparable with DSM-IV-TR defined mixed episode, 15.2%).

Consequently, another consideration is that it could be important to assess, in further studies, the interrater reliability between psychiatrists for the DSM-5 MFS, looking at their low rates of agreement in diagnosis of bipolar and depressive disorders in DSM-5.

As for the prognostic implications, the results are still inconclusive. McIntyre and colleagues [15] underlined the fact that individuals with MDD-MFS exhibited a significantly more severe depression than did individuals with MDD without MFS (adjusted *P* = 0.0002 and *P* < 0.0002, resp.) or BD-MDE without MFS (adjusted *P* = 0.0001 and *P* < 0.0001, resp.).

Finally, individuals with MFS exhibited a higher rate of alcohol/substance use disorder in the context of BD [15] and a younger age at onset [20, 21], as stated in previous researches based on DSM-IV-TR [29–31] meaning that some clinical presentations and disorders in comorbidity are intrinsic to the psychopathology of mixed states and do not depend on the classification used in making a diagnosis.

## 5. Conclusions

The effects and clinical implications associated with the use of the DSM-5 MFS have yet to be fully assessed. Most of the research reports considered in our review were retrospective or used proxies for MFS. Systematic, prospective assessment of mixed symptoms in large population-based cohorts of individuals with MDD or BD was required to understand the meaning and the utility of these symptoms in mood disorders [18]. Furthermore, it is important to ascertain which of the associated features, such as anxiety and substance abuse, are symptomatic of the mixed state itself and which might be amenable to avert poor prognostic outcomes [13].

The identification of additional patients with mixed features according to the updated DSM-5 compared to the DSM-IV-TR definition affirms that the separation of these subtypes of mood episodes from manic or depressive states is necessary in clinical settings mainly to enable the selection of the most effective treatments [8].

Even though the DSM-5 approach considers mixed states as subtypes of manic or depressive episodes [5], mixed depression and mixed mania deserve their own diagnostic identity [4]. In fact, a diagnostic category should be preferred to a specifier, because it will increase focus on mixed states [11], which should be better considered as a spectrum of states backed by the gradation from typical depressive symptoms to typical manic symptoms [19], bringing the current notion of mixed states back to what has been argued by Kraepelin [1] and Akiskal [24].

On the basis of the papers considered in this review, the authors state that the current data are not conclusive; in fact, it is not clear how this new classification can impact the bipolar-unipolar dichotomy and diagnostic reliability, mainly because of the new possibility to diagnose major depression with mixed features [7]. Consequently, further research is needed in order to better understand the implications of the new nosology for epidemiology, clinical care, prognosis, and psychopharmacological treatment of bipolar and unipolar depressed patients that show mixed states features.

It would be important to assess if manic/hypomanic or depressive episodes with mixed features would represent homogeneous groups; maybe, the homogeneity of the different groups can be just explained by the exclusion from the MFS of those manic and depressive symptoms that overlap, such as psychomotor agitation, irritability, and distractibility. Besides, this is the major criticism to the new classification, so its validity needs to be assessed in clinical studies.

In addition, it could be useful to identify the neurobiological, neurocognitive, or neuroimaging correlates that differentiate the different episodes with or without mixed features.

For example, the possibility to separate MDD with and without MFS might help in both identifying patients with worse prognosis and prescribing a more appropriate therapy [7].

It is known that higher rates of suicidal ideation as well as higher frequencies of antidepressant use can be found in bipolar patients with mixed features [32]. As a consequence, studies need to be done in order to assess the increased suicidality in manic/hypomanic and depressed patients with MFS compared to those without MFS, both in bipolar patients and in MDD patients. In fact, this could allow for supporting the recommendation to avoid antidepressant treatment in these patients unless strictly in tandem with mood stabilisers [33] or antipsychotic medications [34].

Furthermore, mood stabilizers and atypical antipsychotics are recommended to treat mixed episodes [34] but data is limited to subanalyses or post hoc analyses of populations of patients with both manic and mixed episodes [7]. Consequently, it would be helpful to involve in randomized prospective trials homogeneous cohorts of mixed patients [34], divided according to the new DSM-5 classification, in order to assess remission rates and prophylactic benefits of the most important mood stabilizers and antipsychotics. This could be innovative mainly in consideration of the fact that patients suffering from MDD with or without MFS can be separated and this can help to distinguish patients that could respond to adjunctive antipsychotics or mood stabilizers from those who would not [7].

At the epidemiological level, the lifetime prevalence of both bipolar disorders and MDD would change and this could be due to the fact that the new MFS could decrease the prevalence of bipolar disorder not otherwise specified (BD-NOS), increasing the prevalence of the specific episodes; this data should be compared to those existing data on mixed states, based on the previous classifications, in order to evaluate the possible differences on epidemiology, morbidity, response to treatment, illness course, and outcomes. In addition, the comparison with this previous data could be useful to ascertain the validity and clinical utility of the new DSM-5 classification [7].

## References

[1] Goodwin F., Jaminson K. (2007). *Manic Depressive Illness*.

[2] Salvatore P., Baldessarini R. J., Centorrino F. (2002). Weygandt's on the mixed states of manic-depressive insanity: a translation and commentary on its significance in the evolution of the concept of bipolar disorder. *Harvard Review of Psychiatry*.

[3] Leonhard K. (1957). *Distribution of Endogenous Psychoses and their Differentiated Aetiology*.

[4] Koukopoulos A., Sani G., Ghaemi S. N. (2013). Mixed features of depression: why DSM-5 is wrong (and so was DSM-IV). *British Journal of Psychiatry*.

[5] Perugi G., Quaranta G., Dell’Osso L. (2014). The significance of mixed states in depression and mania. *Current Psychiatry Reports*.

[6] Verdolini N., Agius M., Quartesan R., Elisei S. (2014). Mixed States: a ‘new’ nosographic entity. *Psychiatria Danubina*.

[7] Vieta E., Valentí M. (2013). Mixed states in DSM-5: implications for clinical care, education, and research. *Journal of Affective Disorders*.

[8] McIntyre R. S., Tohen M., Berk M., Zhao J., Weiller E. (2013). DSM-5 mixed specifier for manic episodes: evaluating the effect of depressive features on severity and treatment outcome using asenapine clinical trial data. *Journal of Affective Disorders*.

[9] Koukopoulos A., Sani G. (2014). DSM-5 criteria for depression with mixed features: a farewell to mixed depression. *Acta Psychiatrica Scandinavica*.

[10] Uher R., Payne J. L., Pavlova B., Perlis R. H. (2014). Major depressive disorder in DSM-5: implications for clinical practice and research of changes from DSM-IV. *Depression and Anxiety*.

[11] de Dios C., Goikolea J. M., Colom F., Moreno C., Vieta E. (2014). Bipolar disorders in the new DSM-5 and ICD-11 classifications. *Revista de Psiquiatría y Salud Mental*.

[12] Liu X., Jiang K. (2014). Should major depressive disorder with mixed features be classified as a bipolar disorder?. *Shangai Archives of Psychiatry*.

[13] Castle D. J. (2014). Bipolar mixed states: still mixed up?. *Current Opinion in Psychiatry*.

[14] First M. B. (2011). DSM-5 proposals for mood disorders: a cost-benefit analysis. *Current Opinion in Psychiatry*.

[15] McIntyre R. S., Soczynska J. K., Cha D. S. (2015). The prevalence and illness characteristics of DSM-5-defined ‘mixed feature specifier’ in adults with major depressive disorder and bipolar disorder: results from the International Mood Disorders Collaborative Project. *Journal of Affective Disorders*.

[16] Malhi G. S., Lampe L., Coulston C. M., Tanious M., Bargh D. M., Curran G. (2014). Mixed state discrimination: a DSM problem that won't go away?. *Journal of Affective Disorders*.

[17] Vieta E., Grunze H., Azorin J.-M., Fagiolini A. (2014). Phenomenology of manic episodes according to the presence or absence of depressive features as defined in DSM-5: results from the IMPACT self-reported online survey. *Journal of Affective Disorders*.

[18] Perlis R. H., Cusin C., Fava M. (2014). Proposed DSM-5 mixed features are associated with greater likelihood of remission in out-patients with major depressive disorder. *Psychological Medicine*.

[19] Takeshima M., Oka T. (2015). DSM-5-defined ‘mixed features’ and Benazzi's mixed depression: which is practically useful to discriminate bipolar disorder from unipolar depression in patients with depression?. *Psychiatry and Clinical Neurosciences*.

[20] Tohen M., Kanba S., McIntyre R. S., Fujikoshi S., Katagiri H. (2014). Efficacy of olanzapine monotherapy in the treatment of bipolar depression with mixed features. *Journal of Affective Disorders*.

[21] Tohen M., McIntyre R. S., Kanba S., Fujikoshi S., Katagiri H. (2014). Efficacy of olanzapine in the treatment of bipolar mania with mixed features defined by DSM-5. *Journal of Affective Disorders*.

[22] Hergueta T., Weiller E. (2013). Evaluating depressive symptoms in hypomanic and manic episodes using a structured diagnostic tool: validation of a new Mini International Neuropsychiatric Interview (M.I.N.I.) module for the DSM-5 'With Mixed Features’ specifier. *International Journal of Bipolar Disorders*.

[23] Zimmerman M., Chelminski I., Young D., Dalrymple K., Martinez J. H. (2014). A clinically useful self-report measure of the DSM-5 mixed features specifier of major depressive disorder. *Journal of Affective Disorders*.

[24] Akiskal H. S. (1996). The prevalent clinical spectrum of bipolar disorders: beyond DSM-IV. *Journal of Clinical Psychopharmacology*.

[25] Cavanagh J., Schwannauer M., Power M., Goodwin G. M. (2009). A novel scale for measuring mixed states in bipolar disorder. *Clinical Psychology and Psychotherapy*.

[26] Henry C., M'Bailara K., Mathieu F., Poinsot R., Falissard B. (2008). Construction and validation of a dimensional scale exploring mood disorders: MAThyS (Multidimensional Assessment of Thymic States). *BMC Psychiatry*.

[27] Benazzi F. (2003). Bipolar II depressive mixed state: finding a useful definition. *Comprehensive Psychiatry*.

[28] Angst J., Gamma A., Benazzi F., Ajdacic V., Rössler W. (2009). Does psychomotor agitation in major depressive episodes indicate bipolarity?. *European Archives of Psychiatry and Clinical Neuroscience*.

[29] Cassidy F., Carroll B. J. (2001). The clinical epidemiology of pure and mixed manic episodes. *Bipolar Disorders*.

[30] Valentí M., Pacchiarotti I., Rosa A. R. (2011). Bipolar mixed episodes and antidepressants: a cohort study of bipolar I disorder patients. *Bipolar Disorders*.

[31] González-Pinto A., Aldama A., Mosquera F., Gómez C. G. (2007). Epidemiology, diagnosis and management of mixed mania. *CNS Drugs*.

[32] Dilsaver S. C., Chen Y.-W., Swann A. C., Shoaib A. M., Krajewski K. J. (1994). Suicidality in patients with pure and depressive mania. *American Journal of Psychiatry*.

[33] Vieta E. (2005). The treatment of mixed states and the risk of switching to depression. *European Psychiatry*.

[34] Fountoulakis K. N., Kontis D., Gonda X., Siamouli M., Yatham L. N. (2012). Treatment of mixed bipolar states. *International Journal of Neuropsychopharmacology*.

